# Draft Genome Sequence of the Steroid Degrader *Rhodococcus ruber* Strain Chol-4

**DOI:** 10.1128/genomeA.00215-13

**Published:** 2013-05-16

**Authors:** Laura Fernández de las Heras, Sergio Alonso, Antonio de la Vega de León, Daniela Xavier, Julián Perera, Juana María Navarro Llorens

**Affiliations:** Department of Biochemistry and Molecular Biology I, Universidad Complutense de Madrid, Madrid, Spaina;; Institute of Predictive and Personalized Medicine of Cancer (IMPPC), Barcelona, Spainb;; Department of Life Science Informatics, B-IT, LIMES Program Unit Chemical Biology and Medicinal Chemistry, Rheinische Friedrich-Wilhelms-Universität, Bonn, Germanyc;; GARP (Genomic and RNA Profiling Core), Dept. Molecular and Human Genetics, Baylor College of Medicine, Houston, Texas, USAd

## Abstract

The whole-genome shotgun sequence of *Rhodococcus ruber* strain Chol-4 is presented here. This organism was shown to be able to grow using many steroids as the sole carbon and energy sources. These sequence data will help us to further explore the metabolic abilities of this versatile degrader.

## GENOME ANNOUNCEMENT

Rhodococci are aerobic, catabolically versatile soil bacteria with a high GC content and high levels of mycolic acids in their cell envelopes. They have been used as model organisms for numerous applications in bioremediation and biocatalysis due to their exceptional ability to degrade a broad range of compounds (1–5). *Rhodococcus ruber* strain Chol-4 was isolated from a sewage sludge sample and it is able to grow in minimal medium supplemented with different steroids as sole carbon sources, such as cholesterol, cholestenone, testosterone, 4-androstene-3,17-dione (AD), 1,4-androstadiene-3,17-dione (ADD), and pregnenolone, among others (6, 7).

In order to get a panoramic view of the genetic complexity and metabolic diversity of this strain, the *R. ruber* strain Chol-4 genome was sequenced using the Roche/454 GS FLX system and assembled with the Newbler assembler version 2.5p1 software (454 Life Sciences). The whole-genome shotgun (WGS) sequence generated 247,832 reads, 89,687,404 bp, that were assembled into 158 contigs with an N_50_ length of 83,586 bp, and an average coverage of 16.3 reads/bp. This contig-assembling process generated 5.4 Mb of data with a G+C percentage of 70.6%. When analyzed with the Glimmer software 3.0 version, using all the coding sequences from the already sequenced *R. equi*, *R. erythropolis*, *R. jostii*, and *R. opacus*, these data revealed 3 rRNA genes, 64 tRNAs genes, and 5,338 putative open reading frames (ORFs).

According to RAST version 4.0 (8) and the Glimmer server, many of these ORFs encode proteins likely involved in the metabolism of different compounds, including amino acids and derivatives (537 ORFs), carbohydrates (472 ORFs), fatty acids/lipids (322 ORFs), proteins (207 ORFs), DNA (110 ORFs), aromatic compounds (100 ORFs), RNA (112 ORFs), sulfur (36 ORFs), phosphorus (33 ORFs), nitrogen (30 ORFs), potassium (20 ORFs), iron (20 ORFs) or secondary metabolic constituents (8 ORFs), among others, while cofactors, vitamins, prosthetic groups, or pigments account for 427 ORFs. The genome also contains 315 ORFs involved in transport, 74 of them being ATP-binding cassette (ABC) transporters (e.g., iron, manganese or molybdate ABC transporters). A total of 104 oxygenase-coding genes acting on aromatic compounds were also identified. This bacterium also contains copper, cobalt, zinc, cadmium, mercuric, arsenic, vancomycin, fluoroquinolone, and beta-lactamase resistance genes.

On the basis of these results, we anticipate that *R. ruber* strain Chol-4 will display a rich and complex metabolic diversity, far beyond the steroid metabolism we have originally found in this strain.

### Nucleotide sequence accession numbers.

This whole-genome shotgun project has been deposited at DDBJ/EMBL/GenBank under the accession number ANGC00000000. The version described in this paper is the first version, ANGC01000000.
